# Whole Blood versus Plasma Samples—How Does the Type of Specimen Collected for Testing Affect the Monitoring of Cytomegalovirus Viremia?

**DOI:** 10.3390/pathogens11111384

**Published:** 2022-11-19

**Authors:** Mateusz Rzepka, Dagmara Depka, Eugenia Gospodarek-Komkowska, Tomasz Bogiel

**Affiliations:** 1 Department of Microbiology, Ludwik Rydygier Collegium Medicum in Bydgoszcz, Nicolaus Copernicus University in Toruń, 85-094 Bydgoszcz, Poland; 2 Department of Clinical Microbiology, Antoni Jurasz University Hospital No. 1, 85-094 Bydgoszcz, Poland

**Keywords:** CMV, CMV DNAemia, cytomegalovirus, molecular diagnostics, plasma, viremia monitoring, whole blood

## Abstract

Viral infections, or their reactivations, are one of the most important groups of transplantation complications that can occur among recipients of both hematopoietic cells and solid organ transplants. They are the most commonly caused by cytomegalovirus (CMV). Currently, the use of whole blood or plasma samples is recommended for CMV viral load monitoring. The aim of the study was to assess and compare the level of CMV DNA, depending on the type of clinical material—whole blood or plasma fraction derived from the same patient. The studies were carried out on 156 whole blood samples in which the presence of CMV genetic material was confirmed and the corresponding plasma samples from the same rounds of sampling. CMV DNA was not present in 59 (37.8%) of plasma samples compared to whole blood-positive counterparts. Of the samples positive in both types of clinical specimen, 77 (79.4%) had higher viral DNA levels in the whole blood samples. There were statistically significant differences in the detected CMV DNA load in the whole blood compared to plasma fraction counterparts (*p* < 0.001). The detected CMV DNA value is usually higher in whole blood compared to plasma samples of the same patient. Due to the variability in CMV viral load depending on the clinical material used for a particular patient, one type of specimen should be always used consequently for CMV viremia monitoring.

## 1. Introduction

Human herpesvirus type 5 (HHV-5), commonly referred to as cytomegalovirus (CMV), belongs to the *Herpesviridae* family, *Betaherpesviridae* subfamily. It belongs to the enveloped viruses and its genome consists of double-stranded DNA enclosed in a capsid structure [1]. This virus is widely distributed in human population and the environment. CMV infections appear most commonly in childhood, but might also be of congenital origin [2,3]. It is estimated that the proportion of CMV-seropositive individuals ranges from 40% to over 90%, depending on the geographic region [2].

CMV transmission occurs often through direct contact with certain secretions (e.g., saliva) or human excretions (e.g., urine). It is also possible for newborns to become infected by breastfeeding from an infected mother [4,5]. Other possible routes of transmission include: sexual contact, blood transfusion, and organ transplantation [6,7].

CMV exhibits a relatively broad cell tropism. This virus is capable of lytic replication, among the epithelial, endothelial, muscle cells and fibroblasts (e.g., skin and lungs). This allows CMV to infect almost all tissues and organs in the human organism [8]. CMV DNA might also be found in the urine, saliva and milk, indicating its release from the site of infection [9].

In immunocompetent individuals, CMV infections are usually asymptomatic or may involve mononucleosis-like syndrome in some cases. Non-specific symptoms may then appear, such as: fever, weakness, muscle pain, enlarged lymph nodes or hepatosplenomegaly [3,7,10].

CMV, like other herpesviruses, is capable of causing latent infections. Persistence is understood as the presence of the CMV genome in the absence of transcription of most viral lytic genes. Re-entry of the virus into the lytic cycle causes the reactivation of infection [11]. In latent form, it occurs primarily in hematopoietic progenitor cells CD34+ and CD33+. This infection is transmitted through the myeloid cells lineage; hence the latent form is not detected in the leukocytes fractions. Thus, it also explains the presence of CMV DNA in monocytes, macrophages and dendritic cells [1,11,12].

The immunosuppressive treatment and lowered immunity, in AIDS patients or allogeneic transplant recipients particularly, predispose to reactivation of a latent infection [13,14,15].

Viral infections constitute one of the most important groups of complications that can occur in recipients of hematopoietic cells and patients after solid organ transplantation most often caused by CMV [16,17,18]. Primary CMV infection or its reactivation in immunosuppressive patients may cause, inter alia, pneumonia, hepatitis, retinitis, as well as enteritis [17]. CMV infection has also been associated with the incidence of graft versus host disease (GvHD) [19]. Other complications include an increase in the incidence of bacterial and fungal infections, which results in antimicrobial treatment with its possible toxic effects. CMV reactivation is also associated with higher mortality in patients with haematological diseases [18]. It has been shown that direct (specific organ damage) or indirect effects (e.g., increased risk of infections, GvHD) of infection have a significant impact on the clinical condition of the recipient and a transplanted organ itself. For this reason, a reliable diagnostic of infections of this etiology is essential [16,17,20].

Primarily, serological and molecular biology methods are used in the diagnosis of CMV infections [16,17,21]. On the other hand, the “gold standard” in the diagnosis of tissue invasive (“end organ”) disease is histological testing with immunohistochemistry [16,22]. The detection of IgG antibodies directed against CMV was used only in the determination of the serological status of donors and recipients. These tests are important in the selection of an antiviral prophylaxis and may influence the decision on an anticipatory treatment administration. They are not useful in the evaluation of active viral replication. Therefore, at present, the detection of IgM antibodies is not recommended for this purpose [16]. Quantitative methods, based on nucleic acid amplification, are recommended for the detection of primary or reactivation of latent infections. They are also used to monitor the effectiveness of antiviral and an immunosuppressive therapy [16,17]. Currently, CMV DNAemia results are reported in IU/mL, in accordance with the international standard introduced by the World Health Organization (WHO) [23]. While this has improved the consistency of the results obtained in different laboratories, there are still many factors that may influence the uniformity of the obtained results. Most often, these differences result from the applied DNA extraction method and the assays used (e.g., limit of detection, specificity, sensitivity, amplification target, amplicon size, used fluorescent probes) [23].

The currently recommended specimens for the CMV DNA presence monitoring are whole blood and plasma [16,17]. As mentioned before, in whole blood samples, CMV is mainly found in monocytes. In contrast, plasma shows predominantly free, highly fragmented CMV DNA, which is released from infected cells [24,25]. However, there is a constant need to determine the degree of correlation between the values obtained from these types of specimens. Therefore, the aim of the study was to assess the level of CMV DNA depending on the clinical material used—whole blood or plasma samples. To the best of our knowledge, this is the first application of the GeneProof PathogenFree DNA Isolation Kit (GeneProof, Brno, Czech Republic) and the GeneProof Cytomegalovirus (CMV) PCR Kit (GeneProof, Brno, Czech Republic) for this purpose.

## 2. Materials and Methods

### 2.1. Clinical Samples

The research was performed on 156 whole blood samples collected in BD Vacutainer^®^ K_3_ EDTA tubes (Becton Dickinson, East Rutherford, NJ, USA). This clinical material was obtained from 53 patients at a primary risk of a latent CMV infection reactivation and was collected from the selected patients several times (up to 13 samples per patient) at intervals. The study group consisted of children with hematological diseases (*n* = 23, 43.4%) and adults after or before solid organ transplantation *(n* = 30, 56.6%) (Table 1). All the samples have been submitted for a routine diagnostics purpose to the Department of Clinical Microbiology of Antoni Jurasz University Hospital No. 1 in Bydgoszcz, Poland. This study was approved by the Bioethics Committee of the Nicolaus Copernicus University in Toruń Collegium Medicum in Bydgoszcz (Approval Code: KB 239/2021, granted on 23 March 2021).

### 2.2. DNA Extraction

**Figure 1:**
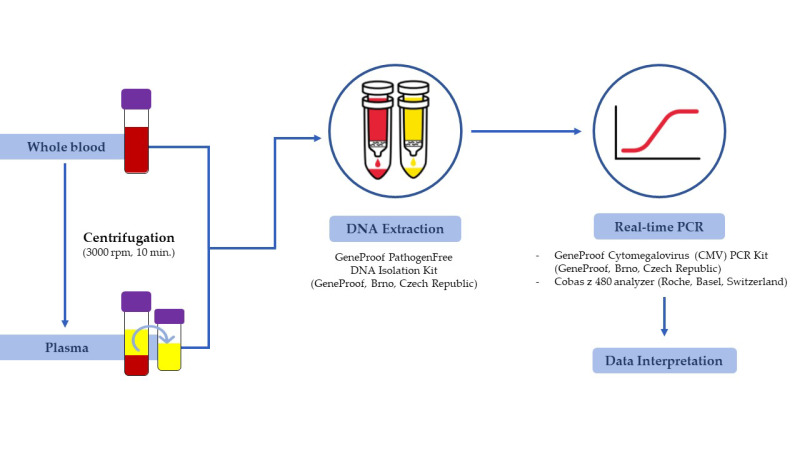
Project design.

In the first stage of the study, DNA was manually isolated from all 156 whole blood samples using the GeneProof PathogenFree DNA Isolation Kit (GeneProof, Brno, Czech Republic) in accordance with the manufacturer’s instructions (Figure 1).

In the second stage of the study, the same whole blood samples were centrifuged (3000 rpm, 10 min.) to obtain plasma fraction. Then the plasma was separated into a sterile tube and the same DNA isolation protocol was applied, as described above (Figure 1). The final volume of the eluates was 100 µL in each case.

### 2.3. Nucleic Acid Amplification Test

Immediately after DNA isolation from the whole blood and plasma samples, two separate real-time PCR reactions were performed using the GeneProof Cytomegalovirus (CMV) PCR Kit - ISEX Version (GeneProof, Brno, Czech Republic) and the Cobas z 480 instrument (Roche, Basel, Switzerland). The reaction conditions were applied according to the manufacturer’s recommendations. The PCR assay has an analytical specificity of 100% (for CMV DNA) and a detection limit of 122.594 IU/mL.

The scheme of the conducted study is shown in Figure 1.

### 2.4. Data Interpretation and Statistical Analysis

The results were interpreted according to the manufacturer’s recommendations. Amplification curves of a specific shape observed at the FAM detection channel (for specific conservative DNA sequence of a single copy gene encoding the exon 4 immediate-early antigen) and simultaneously at the HEX channel (for internal control) were considered as positive results. For the quantification of viremia, a calibration curve was created, and the formula provided by the manufacturer in the kit instructions was applied. The results were presented in the international units per milliliter (IU/mL), according to the standards adopted by the WHO [23].

Statistical analysis was performed in the StatSoft, Inc., Cracow, Poland (2017) STATISTICA 13.1 (data analysis software system, Poland) program using Wilcoxon signed-rank test with *p* < 0.001 to determine the significance of the differences observed between viral load values obtained from the whole blood and the corresponding plasma samples. Spearman’s correlation coefficient was used to assess the correlation of the IU/mL values obtained from the plasma and the corresponding whole blood samples. The standard chi square test was used to determine the significance of the differences observed between the positive and negative results in terms of patients’ age, sex and the initial hospitalization reasons/clinical procedures applied.

## 3. Results

**Figure 2:**
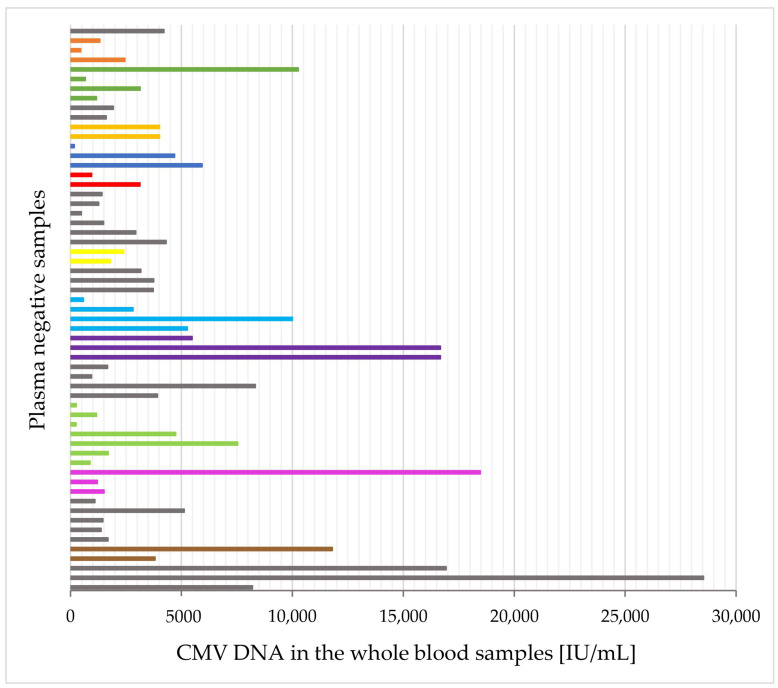
The distribution of CMV DNA load [IU/mL] in the whole blood for the corresponding negative plasma samples (*n* = 59). Individual samples from different patients are marked in gray (one bit represents one sample from one patient). Patients from whom several samples were collected at intervals were marked with a color other than gray (each bit of a given color represents a different sample from the same patient).

Out of 156 whole blood samples with CMV DNA-positive status, CMV DNA was not present in 59 (37.8%) of plasma counterparts (Table 2). As many as 15 (25.4%) of them presented relatively high CMV viral loads in the whole blood (>5000 IU/mL) (Figure 2). Of the plasma negative samples, 10 (16.9%) had whole blood CMV DNA level lower than <1000 IU/mL (Figure 2).

None statistically significant differences were noted between a negative result obtained from plasma and: patients’ sex (χ² = 0.28, *p* = 0.5945) or the initial hospitalization reason (χ² = 0.10, *p* = 0.7550), as compared to the results obtained for the whole blood samples (Table 4).

**Figure 3:**
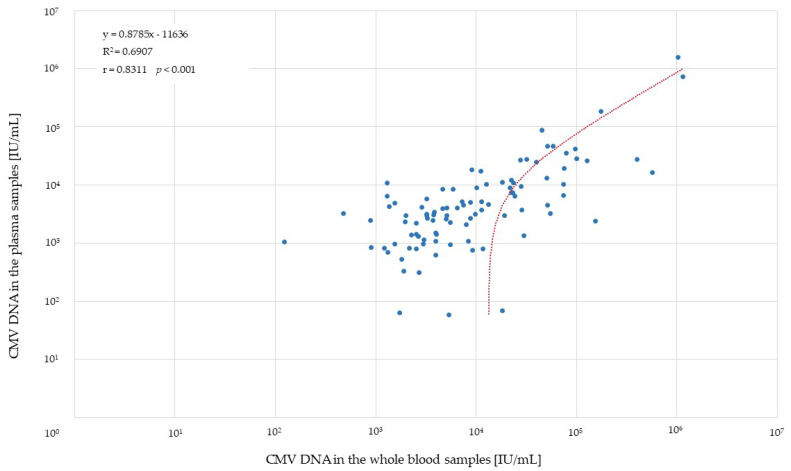
CMV DNAemia [IU/mL] distribution in the whole blood versus the corresponding plasma samples (*n* = 97). The red line represents the regression curve.

Among the samples positive in both types of clinical specimen (*n* = 97) (Table 2), 77 (79.4%) had higher viral load when the whole blood samples were used for the evaluation. As many as 12 (12.4%) of these samples showed over ten times higher values of CMV DNA levels in whole blood than in plasma (Figure 3).

There were statistically significant differences in the number of CMV DNA load detected in whole blood samples, compared to their plasma counterparts (Z = 8.2276, *p* < 0.001). The median DNAemia values for the whole blood samples were also higher than for plasma, 4260 IU/mL versus 1105 IU/mL (Table 4).

Of the 97 (62.2%) samples positive in both (whole blood and plasma), 20 (20.6%) had higher plasma CMV DNA levels. These samples were collected from 13 (24.5%) patients altogether (Table 3).

There was a moderate correlation between the positive results obtained for the whole blood and the corresponding plasma samples (Spearman’s *r* = 0.67, *p* < 0.001) (Table 4).

There was no correlation between a higher value of CMV DNAemia in plasma and sex (χ² = 0.62, *p* = 0.4309) or clinical procedure applied during hospitalization (χ² = 0.51, *p* = 0.4768) as compared to the whole blood samples (Table 4).

## 4. Discussion

CMV infections are an important clinical problem in transplantology [16,17,18]. Hence, it is essential to use reliable molecular diagnostics to assess the actual viral load of this virus. Quantitative molecular tests are currently suggested CMV DNA monitoring in blood or plasma while the detection of pp65 antigen for this purpose is not recommended, due to its insufficient standardization and relatively low sensitivity [16,17,21]. Other clinical material, e.g., urine or saliva, is mainly used to diagnose congenital CMV infection in neonates [26]. Although there are attempts to establish a correlation between systemic infection and the presence of CMV DNA in saliva or urine, this mainly applies to renal transplant recipients [27].

In our study, CMV DNA levels obtained from the whole blood and plasma samples using GeneProof Cytomegalovirus (CMV) PCR Kit (GeneProof, Brno, Czech Republic) were compared. We have shown that the CMV DNAemia is usually higher in whole blood samples, compared to their plasma counterparts. There was also a moderate degree of correlation between the positive results obtained from these two types of clinical material. Similar results were obtained by Lisboa et al. [28], where the range of CMV copies in one ml was from 400 to 1.6 × 10^8^ for whole blood and from 645 to 6.35 × 10^5^ for plasma counterparts. Our results are in line with those from other studies evaluating the utility of whole blood and plasma specimens, however different assays were used in the mentioned studies than in this research [29,30,31,32,33,34].

The detection of higher CMV DNA values in whole blood samples may be associated with the presence of latent CMV in blood monocytes. It is worth mentioning, in plasma fraction, mainly free viral particles are detected [24,25]. The influence of other cells circulating in the blood, e.g., endothelial giant cells infected with CMV, on the values of the obtained results should also be considered [35].

In contrast, some other studies [36,37,38] have generally obtained higher CMV DNA values in plasma. This may be primarily due to differences in the PCR assays used, and thus in the different sequences and sizes of the amplicons [39]. In addition, some studies used different volumes of DNA extraction material (higher for plasma, lower for whole blood) and/or different final elution volumes (lower for plasma, higher for whole blood), which could have influenced the obtained the final CMV DNAemia values.

An important result of our research is that some plasma samples yield higher CMV DNAemia than the whole blood counterparts. So far, no threshold values have been established for the inclusion of anticipatory therapy against CMV infections in such situations [16,17,21]. However, in this study, in some cases (e.g., patient no. 10., Table 3) the obtained CMV DNA levels in plasma were several times higher, which could influence the therapeutic management. Higher plasma values may indicate active viral replication and release of viral particles from cells. These differences may also be due to a changed hematocrit or lymphocyte counts as they do not contain CMV. Other causes include intravascular haemolysis, which may be influenced by drugs intake, autoimmune diseases, or active infections. However, the mentioned clinical data for the test group were not available in this study.

The possible influence of laboratory errors on the obtained results should also be taken into account, e.g., uncalibrated pipettes, inaccurate pipetting, mixing whole blood samples too quickly or storing them too long. In this study, these errors were eliminated or at least reduced by conducting regular intra-laboratory checks and performance of the entire testing by one person only. Nevertheless, it should be emphasized that these are rather isolated situations and, in most cases comparable values of CMV DNA in plasma and whole blood are obtained, which is also confirmed by other studies [28,40]. Therefore, it seems that these differences are not clinically relevant in most cases. Of note, the rules of proper collection, transport and storage of the material for testing must be strictly monitored each time, in order to exclude the influence of these variables on obtaining not typical (e.g., higher) values of CMV DNA in plasma, compared to whole blood samples.

It is worth noting that in this study as much as 37.8% of negative results were obtained when plasma was used for the testing. Some of these results could be explained by the detection of a latent infection in the whole blood samples exclusively. However, a high percentage (25.4%) of negative results was found despite relatively high DNAemia values in whole blood (>5000 IU/mL or higher). Therefore, the routine use of plasma for the detection of CMV infection (or their reactivation) diagnostics might be of limited usefulness. In such a situation, the possibility of an active intracellular replication of the virus should be considered before releasing virions into the plasma.

In this study, no correlation between the CMV viral load in the plasma, the whole blood samples and the origin of the samples (patients’ sex and the hospitalization reason) was demonstrated. It seems that CMV-negative plasma results, as well as the lower or higher values of CMV DNA, do not depend on these parameters, but only on the individual clinical conditions of the patients. However, this observation requires more detailed research.

The limitations of the study were that only one commercially available test was used to assess CMV DNA levels and that we did not apply whole blood samples negative for CMV DNA to check theirs plasma status. Moreover, we did not focus on the current treatment of the patients included in the study and clinical condition of the patients, but for the purposes of the study it was not of the highest importance. We also did not assess whether the higher values of CMV DNA in a dozen or so among plasma samples were due to viral replication.

Summarizing, we support the idea that due to the variability of the CMV viral load, depending on the clinical material used, it is absolutely necessary to use one type of specimen for its consecutive monitoring for a particular patient [41,42].

Important factors influencing the obtained result are primarily the extraction method and the PCR assay used [23,34]. Using the GeneProof Cytomegalovirus (CMV) PCR Kit (GeneProof, Brno, Czech Republic), the median values for the whole blood samples were altogether higher than for plasma. This observation is of great importance in terms of total CMV DNA level monitoring and very important for making the right therapeutic decisions in the further steps [28]. Results obtained with alternating samples type (whole blood or plasma) could lead to misinterpretation [41,42] — actual viremia underestimation of overestimation. We generally proved that the whole blood shows a higher sensitivity in detecting CMV DNAemia compared to plasma fraction. There is a need for further research in order to determine the usefulness of low CMV DNA levels in whole blood and, at the same time, undetectable in plasma, e.g., in comparison to the clinical condition of the patients. Given the high proportion of CMV seropositive patients in all geographic regions [2], detection of low CMV DNA values may not be clinically relevant (latent infections). On the other hand, low values may alert clinicians to a more frequent CMV DNAemia monitoring for an earlier detection of infection reactivation. Thus, it would enable the rapid implementation of an appropriate treatment and reduce the direct and indirect effects of the active infection [28].

## 5. Conclusions

Since CMV infections are an important clinical problem in transplantology, it is essential to use reliable molecular diagnostics to assess the viral load. The detected CMV DNA level is usually higher in the whole blood compared to plasma samples of the same patient. Due to the variability in CMV viral load, depending on the clinical material used for a particular patient, one type of specimen should be always used consequently for CMV viremia monitoring. This is important in a reliable evaluation of CMV replication kinetics and making the right therapeutic decisions consecutively.

## Figures and Tables

**Table 1:** General characteristics of the patients (*n* = 53).

Age Group	Sex	*n*	Initial Hospitalisation Reason
Children	Female	11	Oncology and/or hematology
	Male	12	
Adults	Female	15	Solid organ transplantation
	Male	15	

**Table 2:** Number and percentage of CMV DNA detection results in the plasma versus the whole blood samples (*n* = 156).

	Whole Blood	Plasma	
	Positive CMV DNA	Positive CMV DNA	Negative CMV DNA
*n*	156	97	59
%	100	62.2	37.8

**Table 3:** The list of CMV DNA levels results [IU/mL] for the samples with greater plasma DNAemia when compared to the whole blood counterparts (*n* = 20).

Patient No.	CMV DNA [IU/mL]	
	in Whole Blood	in Plasma
1.	2870	4155
2.	1530	5000
3.	880	2520
4.	475	3253
5.	3180	5850
	3100	3240
	5850	8500
	1970	3040
6.	1360	4325
7.	9000	18,450
8.	123	1055
9.	172,670	188,000
	1950	2365
10.	1290	10,900
	1320	6550
	4613	8700
11.	1,013,340	1,585,000
	11,070	17,450
12.	27,270	27,300
13.	45,270	88,500

**Table 4:** Summary of statistical results.

Statistical Analysis		Statistically Significance
Correlation of negative plasma result vs.		
- patient sex	χ² = 0.28, *p* = 0.5945	No
- the initial hospitalization reason	χ² = 0.10, *p* = 0.7550	No
Correlation of the number of CMV load in the whole blood vs.		Yes
- the corresponding plasma samples	Z = 8.2276, *p* < 0.001	
Correlation of CMV load in samples positive in both specimens, whole blood and plasma	Spearman’s *r* = 0.67, * p * < 0.001	Yes
Correlation of higher value of CMV load in plasma vs.		
- patient sex	χ² = 0.62, *p* = 0.4309	No
- the initial hospitalization reason	χ² = 0.51, *p* = 0.4768	No
Median DNAemia values for:		
- the whole blood samples	4260 IU/mL	
- the plasma samples	1105 IU/mL	

## Data Availability

The data presented in this study are available on request from the corresponding author.
